# Othering am Beispiel von Migration: Wie aus sozialen Kategorien die Anderen entstehen

**DOI:** 10.1007/s00103-023-03763-8

**Published:** 2023-09-18

**Authors:** Nurcan Akbulut, Oliver Razum

**Affiliations:** 1 AG Epidemiologie & International Public Health, Fakultät für Gesundheitswissenschaften, Universität Bielefeld, Bielefeld, Deutschland; 2 Forschungszentrum gesellschaftlicher Zusammenhalt (FGZ), Standort Bielefeld, Bielefeld, Deutschland

**Keywords:** Othering, ‚Geflüchtete‘/‚Migranten‘, Public Health, Gesundheitliche Ungleichheit, Epistemische Ungleichheit, Othering, ‘Refugees’/‘Migrants’, Public health, Health inequalities, Epistemic inequality

## Abstract

‚Migranten‘ und ‚Geflüchtete‘ werden häufig als Andere kategorisiert in einem Prozess, der als Othering (Veranderung) bezeichnet wird. Am Beispiel von (Flucht‑)Migration entwickeln wir eine Definition des Begriffs Othering, um ihn für die Analyse gesundheitlicher Ungleichheiten nutzbar zu machen. Othering verstehen wir dabei als einen gesellschaftlichen Prozess, der Unterschiede so konstruiert und klassifiziert, dass bestimmte Gruppen als wesentlich Andere sozial sichtbar werden. Dem Prozess des Othering liegt zum einen eine diskursive Praxis zugrunde, die Differenzen markiert und damit Menschen zu sichtbar Anderen macht. Zum anderen beruht er auf einer Machtasymmetrie, die es ermöglicht, Menschen zu kategorisieren und damit als anders zu markieren.

Othering beruht nicht allein auf ablehnenden Einstellungen einzelner Personen oder Gruppen. Vielmehr ist Othering das Resultat eines umfassenden und historisch gewachsenen Systems von Überzeugungen, die durch Machtbeziehungen Glaubwürdigkeit erlangen. Insofern verstehen wir Othering als einen machtvollen Prozess, der über Diskriminierungskonzepte, die auf bloße Kategorisierungsprozesse gründen, wesentlich hinausgeht. Das Othering-Konzept hebt sich von anderen Konzepten der Ungleichheit ab, indem es die epistemische Ebene als wesentlichen Faktor für Ungleichheit einbezieht. Othering erzeugt nicht nur begrifflich die Anderen, sondern begründet zugleich eine diskursive Legitimation für den ungleichen Umgang mit dem Anderen.

Ausgehend von unserem Verständnis von Othering stellen wir praxisbezogene Ergebnisse zu den Auswirkungen des Othering auf die Gesundheitsversorgung von ‚Migranten‘ und ‚Geflüchteten‘ dar.

## Abstract (en)

‘Migrants’ and ‘refugees’ are often categorized as Other, in a process called Othering. Using the example of forced migration, we develop a definition of Othering to make it useful for the analysis of health inequalities. We consider Othering as a social process that constructs and classifies differences in such a way that certain groups become socially visible as essential Others. On one hand, the process of Othering operates through a discursive practice that constructs differences, thereby transforming individuals into visible Others. On the other hand, it is based on a power asymmetry that enables the categorization of people, thereby marking them as different.

Othering is not solely based on negative attitudes of individuals or groups. Rather, Othering is the result of a broad and historically evolved system of beliefs that gain credibility through power relations. Thus, we understand Othering as a powerful process that goes substantially beyond concepts of discrimination based on mere categorization processes. The concept of Othering stands out from other concepts of inequality by including the epistemic level as a key factor for inequality. Othering not only produces the Other epistemologically, but at the same time establishes a discursive legitimation for the unequal treatment of the Other.

Drawing upon our understanding of Othering, we present practice-related findings on the consequences of Othering for the healthcare of ‘migrants’ and ‘refugees’.

## Einleitung

‚Migranten‘^1^ und ‚Geflüchtete‘ werden häufig als Andere kategorisiert, in einem Prozess, der als Othering (Veranderung) bezeichnet wird. Für den Bereich Public Health mangelt es bislang an einer Definition und Abgrenzung dieses Begriffs.^2^ Um ihn für die Analyse gesundheitlicher Ungleichheiten nutzbar zu machen, entwickeln wir zunächst ein konzeptionelles Verständnis^3^ von Othering, das wir exemplarisch anhand von Migration beleuchten. Unser Ausgangspunkt sind poststrukturalistisch-diskurstheoretische und postkoloniale Ansätze. Anschließend präsentieren wir empirische Befunde, welche gesundheitsrelevante Auswirkungen des Othering aufzeigen. Wir schließen mit theoriegeleiteten, definitorischen Merkmalen des Othering ab.

## Unterscheidungskategorien als diskursiv hervorgebrachte Konstrukte

Soziale Differenzkategorien lassen sich aus diskurstheoretischer Perspektive als diskursive Artefakte auffassen [1–3]. Sie sind nicht naturgegeben und basieren nicht auf Tatsachen. Soziale Kategorien repräsentieren nicht unsere Realität, sondern konstituieren diese. Sie sind Konstrukte, die innerhalb von diskursiven Strukturen geformt werden und unsere Wahrnehmung der Wirklichkeit beeinflussen [2, 4]. Um die Welt verstehbar zu machen, gliedern wir sie mithilfe sprachlicher Differenzierungen [5]. Die im Alltag verwendete Sprache hilft, Erfahrungen und Phänomene in klar definierte und verständliche Begriffe oder Kategorien zu übersetzen. Durch diese Objektivierungen entsteht eine Ordnung [3], innerhalb derer differenzierende Begriffe und Kategorien Sinn ergeben. Wir gebrauchen Kategorien, um unsere soziale Realität zu strukturieren, zu interpretieren und damit Ordnung zu stiften [3]. Dabei reduzieren wir allerdings die Komplexität unserer Wirklichkeit.

### Wissens- und Klassifikationssysteme

Soziale Unterscheidungskategorien, die für die Konstruktion der Anderen bedeutsam werden, sind keine beliebigen Konstrukte. Sie entstammen einem diskursiv geprägten Wissens- und Klassifikationssystem [2], das sie als selbstverständlich erscheinen lässt. Jede Epoche – um mit Foucault zu argumentieren – bringt ein charakteristisches Wissens- und Klassifikationssystem hervor. So war es beispielsweise im 19. Jahrhundert, während der Viktorianischen Epoche, üblich, Menschen nach ihrer ‚Rasse‘ und Reinheit ihrer Abstammung zu klassifizieren. Damals wurde der sog. Mischlingscharakter der spanisch-amerikanischen Bevölkerung Perus anhand von 32 Kategorien akribisch definiert und dokumentiert [4].

Die Konstruktion unterschiedlicher ‚Rassen‘ entstand innerhalb eines umfassenden Wissenssystems, sodass es zur Zeit des Viktorianismus als normal und legitim galt, Menschen so zu kategorisieren. In der heutigen Zeit ist es inakzeptabel und entbehrt jeder wissenschaftlichen Grundlage, Menschen nach ihrer vermeintlichen ‚Rassenzugehörigkeit‘ einzuordnen, da diese Betrachtungsweise rassistisches Gedankengut reproduziert.

Diese Argumentation ist von der Vorstellung geprägt, dass *moderne* Gesellschaften sich weiterentwickelt hätten, und ist tief verwurzelt in der europäischen Geschichte, die als Geschichte eines Erkenntnis- und Entwicklungsfortschritts erzählt wird [4]. Die Arbeiten Foucaults jedoch stellen genau dieses Postulat des Fortschritts (die Entwicklung vom Schlechteren zum Besseren) infrage [4]. Foucault [1, 2] untersucht, wie diskursive Systeme sich über die Zeit verändern und wie sich damit auch die jeweilige Perspektive auf die Realität verändert. Für seine Analysen entwickelte er den Begriff der Episteme [1, 2], der sich auf die strukturellen Bedingungen bezieht, unter denen Wissen in verschiedenen historischen Epochen erzeugt wird. Eine Episteme kann als Grundlage des Denkens betrachtet werden, die zu einer bestimmten Zeit bestimmte Aussagen als Wissen akzeptiert und andere nicht. Sie setzt sich aus der Gesamtheit der diskursiven Strukturen zusammen, die aus der Interaktion einer Vielzahl zirkulierender und autorisierter Diskurse entstehen [4]. Foucault identifiziert Diskontinuitäten in Diskursen und verweist auf Veränderungen innerhalb diskursiver Strukturen, die zu einem Wandel im Wissen führen. Er postuliert, dass es nicht primär der Fortschritt ist, der hierfür die Triebkraft ist, sondern die Veränderungen in unseren epistemischen Wissens- und Klassifikationssystemen [1, 2]. Dadurch erscheinen uns die gegenwärtigen Klassifizierungen und Kategorisierungen, an die wir gewöhnt sind, als vollkommen normal und natürlich, so ähnlich wie es Menschen im 19. Jahrhundert normal erschien, nach ‚Rassen‘ zu kategorisieren [4].

Die Geschichte der Moderne ist ein Diskurs des Fortschritts, der ein Gegenmodell benötigt, um sich als fortschrittlich konstruieren zu können. Erst *Rückschritt* und *Unzivilisiertheit* als Entgegensetzung ermöglichen einen Fortschrittsdiskurs [5, 9–11]. In postkolonialen Studien [12–14] dient Othering als zentrale differenzkritische Analyseperspektive, um zu untersuchen, wie die klassischen Modernisierungsdiskurse ihr Gegenmodell produzieren [15–20].

Said [15] und Spivak [16] dekonstruieren westliche Wissenschaftsparadigmen. Ihre postkoloniale Kritik betrachtet das imperialistische Projekt Europas, das die Kolonien und Kolonisierten im Sinne eines Othering als Andere objektivierte [21]. Diese diskursive Hervorbringung und Aneignung des Anderen durch Wissen, von Spivak als „epistemic violence“ [16] bezeichnet, zeigt die Zusammenwirkung von epistemisch-symbolischer und materieller Ebene. Das Othering-Konzept hebt sich von anderen Konzepten der Ungleichheit ab, indem es die epistemische Ebene als wesentlichen Faktor für Ungleichheit einbezieht. Othering erzeugt somit nicht nur begrifflich die Anderen, sondern begründet zugleich eine diskursive Legitimation für den ungleichen Umgang mit dem Anderen – ein Umstand, den wir im Kontext der Migration als epistemische Ungleichheit bezeichnen.

## Zusammenhang zwischen sozialen Unterscheidungskategorien und Othering

**Figure 1:**
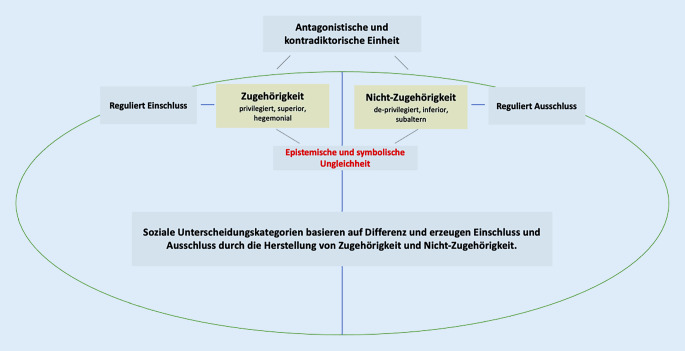

Jede soziale Kategorie, die zur Kennzeichnung von Gruppen dient und zur Aufrechterhaltung einer bestimmten sozialen Ordnung beiträgt – oder noch genauer: die notwendig ist, um die Einheit und Reinheit dieser Ordnung zu bewahren –, beinhaltet eine binäre Logik, quasi einen Widerspruch, und funktioniert durch die Herstellung von Differenz. Daher ist die Rede auch von Differenz- oder Ordnungskategorien. Soziale Differenzkategorien arbeiten mit dem bipolaren Schema von Zugehörigkeit und Nicht-Zugehörigkeit. Sie erzeugen Einschluss und Ausschluss, indem sie Zugehörige von Nicht-Zugehörigen unterscheiden [5]. Zugehörige wie Nicht-Zugehörige stehen in einer abhängigen Beziehung zueinander, denn ohne Nicht-Zugehörige wäre der Begriff der Zugehörigkeit bedeutungslos. Die Setzung von Zugehörigen bewirkt simultan die Ausschließung von Nicht-Zugehörigen und umgekehrt. Das Verhältnis zwischen Zugehörigen und Nicht-Zugehörigen ist nicht gleichberechtigt – es zeichnet sich durch epistemische und symbolische Ungleichheit aus ([7, 15, 16]; Abb. 1).

Dieser wichtige Aspekt lässt sich am Beispiel der Unterscheidung zwischen ‚Migranten‘ und ‚Nicht-Migranten‘ genauer nachvollziehen.

### Othering am Beispiel der Unterscheidung zwischen ‚Migranten‘ und ‚Nicht-Migranten‘

Die Kategorie ‚Migrant‘ entfaltet ihre Bedeutung durch die Unterscheidung und begriffliche Abgrenzung von der Kategorie des ‚Nicht-Migranten‘. Um diese binäre Logik legitimieren und aufrechterhalten zu können, muss diese Unterscheidung semantisch aufgeladen werden: Es müssen Differenzen zwischen ‚Migranten‘ und ‚Nicht-Migranten‘ hergestellt werden, um sie voneinander unterscheidbar machen zu können. Dies vollzieht sich vorwiegend auf einer diskursiven Ebene. Diskurs lässt sich hier vereinfacht als ein gesellschaftlich konventionalisiertes – also ein normatives – Sprechen über in diesem Fall Migration und die durch sie hervorgebrachten Subjekte (‚Migranten‘, ‚Geflüchtete‘ etc.) beschreiben. Der Diskurs als eine Praxis der Bedeutungsproduktion [17] lässt sich nicht allein auf sprachliche Äußerungen reduzieren, sondern er umfasst jegliche Formen der Repräsentation (z. B. auch Museen). Hall spricht in diesem Zusammenhang von Systemen der Repräsentation [17]. Unter Repräsentation versteht er die Markierung und Hervorhebung von Differenz und die temporäre Festschreibung ihrer Bedeutung [17].

In diesem diskursiven Sprechen zeigt sich eine bestimmte Repräsentationsform, die sich auf einer epistemischen Ebene entfaltet und im politischen, medialen, wissenschaftlichen wie auch im Alltagsdiskurs zum Ausdruck kommt. Es ist insofern eine machtvolle Repräsentation, als dass sie institutionalisiert ist [17]. Die diskursive Aufladung der Kategorie des ‚Migranten‘ geschieht durch die diskursive Verknüpfung mit selektiv gewählten Themenbereichen. Dazu gehören Integration, kulturelle Differenz, die sog. Flüchtlingskrise, Gewalt, Verbrechen, Unruhen und andere Formen der Abweichung, die vorwiegend auf Probleme und Konflikte fokussieren [22–24]. Daneben existieren spezifische auf die Anerkennung von Migration abzielende Diskurse, wie beispielsweise Diversität, soziale und gesundheitliche Ungleichheit sowie Diskriminierung. Die diskursiv hergestellten Zusammenhänge unterliegen keiner notwendigen Struktur, jedoch nehmen sie in ihrer repräsentierten Form den Charakter von Wirklichkeit an.

Neben dem Migrationsstatus werden weitere Zugehörigkeitskategorien im Diskurs bedeutsam gemacht, z. B. die nationale und ethnische sowie die kulturelle und religiöse Zugehörigkeit. Mecheril spricht in diesem Zusammenhang von „natio-ethno-kultureller Zugehörigkeit“ [25] und weist auf die intersektionale [26] Struktur der Migrationskategorie hin: Die Kategorie ‚Migrant‘ oder ‚Migrationshintergrund‘ ist keine eindimensionale Kategorie, in ihr sind weitere, miteinander verwobene Differenzkategorien wirksam [27]. Die konstruierten ethnisch-kulturell-religiösen Unterschiede trennen ‚Migranten‘ und ‚Nicht-Migranten‘ [28].

Durch diese eben genannten diskursiven Verknüpfungen werden ‚Migranten‘ nicht nur unterscheidbar gemacht, sondern auch als eine besonders deviante – von der herrschenden Norm abweichende – Gruppe hervorgebracht. Als eine Gruppe, von der eine besondere Gefahr ausgeht [29], die eine besondere Belastung oder Herausforderung darstellt [30], oder auch als eine vulnerable Gruppe, die einer besonderen Förderung bedarf. Das Nicht-Migrantische erscheint in dieser Konstellation als Normalität.

Die semantische Aufladung der Kategorie des ‚Migranten‘ erzeugt keine bloße Dichotomie zwischen ‚Migranten‘ und ‚Nicht-Migranten‘, in der sich zwei unterscheidbare Gruppen gegenüberstehen. Es sind nicht die Gruppenkategorien an sich, sondern vielmehr die diskursive Hervorbringung von Kategorien und deren essentialisierender Gebrauch ([31]; siehe Abschnitt „Othering in der Gesundheitsversorgung und gesundheitswissenschaftlichen Forschung“). Der Diskurs über Migration bringt nicht nur Subjekte als sichtbare Andere (z. B. ‚Geflüchtete‘, ‚Migranten‘) hervor, sondern unterwirft sie auch jenen Kategorien, die sie als anders kennzeichnen. Infolgedessen erscheinen ‚Migranten‘ sowohl in der Betrachtung von ‚Nicht-Migranten‘ als auch in ihrer Selbstwahrnehmung als ‚Migranten‘. Spivak beschreibt diesen Akt der gewaltvollen Unterwerfung im Zusammenhang von Subalternität [32] als Unmöglichkeit, jenseits einer binären Identitätslogik zu sprechen.

Der Subjektivierungsprozess hat eine identitätsstiftende Funktion, da durch die iterative Konstruktion der Anderen simultan ein normatives Selbstverständnis von einem Wir (Nicht-Anderen) entsteht. Die Fixierung auf naturalisierte Unterschiede der Anderen fungiert als Kontrastfolie für die eigene Identitätsbildung, die sich gegenüber dem semantisch aufgeladenen Wissen über die Anderen abgrenzt [33, 34].

Die Unterscheidung zwischen ‚Migranten‘ und ‚Nicht-Migranten‘ als eine gegeben angenommene Wissensform ermöglicht, sowohl unterschiedliche Bevölkerungsgruppen zu kategorisieren und zu unterscheiden als auch alltägliche Praktiken zu strukturieren, und verleiht ihnen eine Bedeutung. Als gesellschaftlich bedeutungsvolle Kategorien manifestieren sich Othering-relevante Unterscheidungen auch in den strukturellen Logiken von Institutionen. Diese können sowohl von Institutionen selbst als auch von Individuen genutzt werden, da sie allgemein zugänglich sind [25]. Gemeint sind damit die diskursiven Verbindungen, die den als migrantisch Markierten auf eine konstitutive Andersheit festlegen und reduzieren. Wobei hier die Festlegung der ‚Migranten‘ weniger auf die Migrationserfahrung zurückzuführen ist (was der Begriff ‚Migrationshintergrund‘ verdeutlicht), sondern vielmehr auf eine „zugeschriebene Abweichung von Normalitätsvorstellungen“ [25], die sich in bestimmten Merkmalen, wie z. B. Habitus, Aussehen, Namen, äußert. Sowohl ‚Migranten‘ als auch ‚Nicht-Migranten‘ entwickeln auf dieser Basis ein Verständnis darüber, wer als ‚Migrant‘ gilt und wie ‚Migranten‘ gesehen, verstanden und behandelt werden sollen. ‚Nicht-Migranten‘ kommt jedoch durch ihre privilegierte Rolle in diesem Kontext eine Machtposition zu, die regulär ‚Migranten‘ vorenthalten bleibt. Die Position der Nicht-Anderen fungiert als ein privilegierter Ort der Beobachtung, Kategorisierung, Klassifizierung und Bewertung. Die Sprecherposition der Anderen hingegen ist mit einer geringeren epistemischen Macht ausgestattet [34].

**Figure 2:**
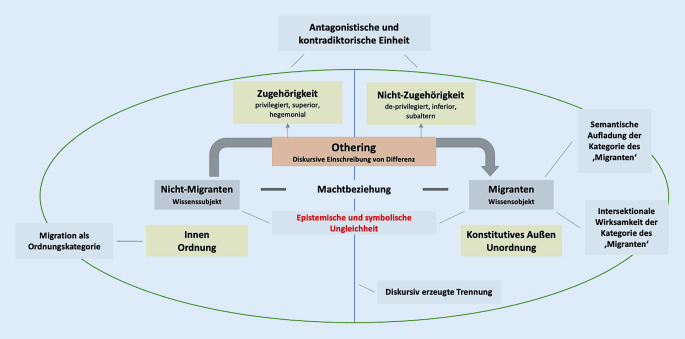

Insofern ruft diese Unterscheidung nicht nur symbolische und epistemische Effekte hervor, die sich in der Hierarchisierung durch Ein- und Ausschluss von Zugehörigkeiten manifestieren, sondern auch materielle Effekte, welche die Verteilung von Macht und Ressourcen beeinflussen. Man kann daher von der Konstruktion der ‚Migranten‘ als die Anderen sprechen, die einem ‚Wir‘ gegenübergestellt werden – in diesem Fall den ‚Nicht-Migranten‘ (Abb. 2).

### Othering als Machtrelation zwischen einem Innen und Außen

Othering verweist nicht nur auf die bloße Konstruktion von Gruppen, Fremdheit oder Andersheit. Durch Othering hervorgebrachtes Wissen basiert nicht auf individuellen Ansichten oder Einstellungen spezifischer Gruppen, wie beispielsweise rechtsextremen, sondern ist das Resultat eines umfassenden und historisch gewachsenen Systems von Überzeugungen, die diskursiv strukturiert sind und durch Machtverhältnisse Glaubwürdigkeit erlangen [4]. Ebenso entscheidend für den Prozess des Othering ist, dass er aus einer antagonistischen Machtbeziehung zwischen den Anderen und Nicht-Anderen hervorgeht und zugleich eine Bedingung als auch eine Folge dieser Machtrelation darstellt. Andere sowie Nicht-Andere sind als relationale Konstruktionen zu verstehen; ihre Bedeutung konstituiert sich aus ihrem dichotomisch konstruierten Verhältnis zueinander, wobei das Nicht-Andere als Normalität etabliert und dadurch normativ wirksam wird [35].

Am Beispiel von ‚Migranten‘ und ‚Nicht-Migranten‘ zeigt sich dieser Antagonismus und damit verbunden die Nicht-Zugehörigkeit von ‚Migranten‘, welche durch das spezifische In-Beziehung-Setzen von nationalstaatlicher Einheit und Migration konstruiert wird [36]. Zwischen diesen beiden Gruppen – ‚Migranten‘ und ‚Nicht-Migranten‘ – besteht ein Exklusionsverhältnis, das eine Trennung^4^ zwischen einem Innen und einem konstitutiven Außen erzeugt [37, 38]. Das Innen symbolisiert eine bestimmte soziale Ordnung, das Außen hingegen das Andere, die Abwesenheit und Negation dieser Ordnung im Sinne einer Unordnung [5, 39]. Das Innen dieser Ordnung ist mit dem, was sie ausschließt, eng verwoben. Das Außen ist konstitutiv für das Innen, da das Eigene relational und supplementär mit dem Anderen verbunden ist. ‚Migranten‘ gehören also zu dieser Ordnung, jedoch als ihr Gegenstück bzw. Anderes [5].

Das Innen und Außen sind keine natürlichen Entitäten und stellen Imaginarien dar, die diskursiv hervorgebracht werden – insofern besitzen sie keine eigenständige unabhängige Existenz [39]. Da die Abgrenzung und Beziehung zum Außen gleichsam die Bedingung der Existenz dieser Ordnung darstellen, werden die Anderen konstitutiv für diese Ordnung und müssen immer wieder reartikuliert werden [39]. Othering entfaltet seine Wirkung durch den Prozess seiner Wiederholung und Wiederholbarkeit. Das bedeutet, die Anderen werden im Verhältnis zu dieser Ordnung repräsentiert und immer wieder als Abweichung von der eigenen geordneten Normalität hervorgebracht. Die Besonderheiten der Anderen werden in einer extremen Form der Abweichung repräsentiert, problematisiert und im Außen lokalisiert – z. B. können sie sich in einer abwertenden oder idealisierenden Tendenz manifestieren oder beides zugleich. Sie werden naturalisiert und essentialistisch gedacht – in der Folge erscheinen sie dann als eine unveränderliche Konstante oder anders formuliert: als Wesensmerkmal der Anderen (Abb. 2).

## Othering in der Gesundheitsversorgung und gesundheitswissenschaftlichen Forschung

Die aus Othering hervorgehende epistemische Ungleichheit in Form von Wissen über die Anderen ist auf allen Gesellschaftsebenen anzutreffen. Auf einer interpersonellen Ebene kann sich das Wissen über die Anderen in den Einstellungen von Individuen äußern und die Gesundheitsversorgung beeinträchtigen. Eine aktuelle Vignetten-Studie zeigt, dass Flüchtlingsstatus, zugeschriebene Religionszugehörigkeit und Geschlecht der Patient*innen einen signifikanten Einfluss auf die Empfehlungen von Psychotherapeut*innen haben. Psychotherapeut*innen neigen dazu, mehr therapiehemmende Einstellungen gegenüber Patient*innen mit muslimischem Namen und Fluchtstatus zu haben. Die Behandlung dieser Gruppe wird mit mehr Schwierigkeiten und negativen Emotionen verbunden, was häufiger zur Ablehnung der Therapie führt. Die Voreingenommenheit steigt, wenn weitere Faktoren wie weibliches Geschlecht hinzukommen, was die intersektionale Ausprägung des Othering verdeutlicht [41].

Othering ist auch auf anderen Ebenen wirksam. Der diskursiv erzeugte Bedrohungscharakter der Anderen legitimiert Sonderregelungen zu ihrer Kontrolle. So können verschärfte Regelungen im Asylbewerberleistungsgesetz und Asylrecht als Strategie interpretiert werden, um steigende Zuwanderungszahlen kontrollierbar zu machen, wobei Einschnitte in die Existenzsicherung hingenommen werden [42]. Sozialräumlich begünstigt die Darstellung Geflüchteter als gesellschaftliche Herausforderung die Legitimierung räumlicher Trennung in Lagern [43, 44]. Die bewusste Degradierung des Wohnraums beschreibt Pieper [44] als staatlich gewollte Abwertung der sozialen Stellung, was öffentliche Stigmatisierung zur Folge hat. Die Unterbringungsart beeinflusst die Gesundheit von Geflüchteten. So ist die psychische Gesundheit von Personen in privaten Unterkünften signifikant besser als in Gemeinschaftsunterkünften [45].

Auch die Gesundheitsforschung trägt durch Kategorisierungs- und Klassifizierungsmethoden zur Konstruktion von ‚Migranten‘ als Andere bei. Beispielsweise verwendete die deutsche Gesundheitsberichterstattung in den 1980er-Jahren die Kategorie ‚Ausländer‘, um ‚Migranten‘ im Rahmen der Tuberkulose-Debatte als epidemiologisches Problem darzustellen [46]. Eine kategoriale Unterscheidungspraxis ist aus epistemologischer Sicht kritisch zu betrachten, da sie die Präexistenz sozialer Gruppen unterstellt. Durch kontinuierliche Wiederholung und Bestätigung erscheinen soziale Unterscheidungskategorien als natürliche Kategorien. Dies gilt insbesondere für soziale Ordnungskategorien wie Migration oder Ethnizität, welchen eine inhärente Natürlichkeit zugesprochen wird und die als individuell nicht modifizierbar gelten [47]. In ihrer Konsequenz führen diese Kategorien zur Verobjektivierung von ‚Migranten‘. Daher wird empfohlen [48], soziale Kategorisierungen, wie etwa den ‚Migrationshintergrund‘, in amtlichen und wissenschaftlichen Datenerhebungen ausschließlich zur Untersuchung von Diskriminierung zu nutzen.

## Schlussfolgerungen zu einer Definition des Othering

Wenn wir die theoretischen Überlegungen in einen Definitionsansatz für Othering integrieren, lassen sich folgende gleichzeitig wirkende Merkmale identifizieren:

*Othering basiert auf epistemischer Ungleichheit und operiert als diskursive Unterscheidungspraxis. Durch Verobjektivierung von Differenz bei der Wissensproduktion werden bestimmte Gruppen als Andere in Abgrenzung zu einem Wir sozial sichtbar.*

Othering ist ein Konstruktionsprozess, der Individuen in Kollektivkategorien einteilt und eine hierarchische Anordnung von Zugehörigkeiten durch regulierten Ein- und Ausschluss schafft. Die Kategorie des Anderen ist intersektional mit anderen Kategorien verschränkt. Die semantische Aufladung von Zugehörigkeitskategorien und Differenzen entsteht durch diskursive Wissensproduktion und Verobjektivierung, die auf epistemischer Ungleichheit basieren und diese Ungleichheit aufrechterhalten. Zum Beispiel ist es ein Privileg, als ‚Nicht-Migrant‘ klassifiziert zu werden, da diese Kategorie nicht markiert ist und keine spezifische semantische Aufladung hat. Die Perspektive auf ‚Nicht-Migranten‘ ist differenzierter, während die Figur des ‚Migranten‘ durch ihre spezifische semantische Aufladung präfiguriert ist und dadurch hohe soziale Sichtbarkeit erlangt.

*Othering erzeugt und verstetigt eine Dominanzrelation zwischen Anderen und Nicht-Anderen und führt zu Ungleichheit in verschiedenen Dimensionen und auf mehreren Ebenen.*

‚Migranten‘ erfahren auf verschiedenen Ebenen und entlang verschiedener Kategorien (Geschlecht, Migrationsstatus, Religion, Hautfarbe etc.) eine Ungleichbehandlung. Dies äußert sich z. B. darin, dass vorhandene Hierarchien (z. B. zwischen Ärzt*in und Patient*in) durch Othering verschärft oder umgekehrt werden.

*Othering ist identitätspolitisch wirksam und erzeugt ein projektives Gegenbild zum Selbstbild.*

Die Anderen stellen ein komplementäres und projektives Gegenbild zum Selbstbild dar. Das verborgene oder nach außen propagierte Selbstverständnis des Nicht-Anderen determiniert das Verhältnis zum Anderen. Durch den Ausschluss der Anderen kann ein Zusammenschluss des Wir als exklusive und homogene Gruppe hergestellt bzw. forciert werden. In ihren Abweichungen bestätigen die Anderen die Selbstverständlichkeiten und die Normalität des Wir. Das Nicht-Andere und das Andere werden als feststehende Entitäten gesetzt, denen eine natürliche Erscheinung zugeschrieben wird.

*Othering erzeugt durch Subjektivierung und Unterwerfung sich selbst konsolidierende Andere und erzeugt Subalternität.*

Die diskursive Hervorbringung und Unterwerfung von Subjekten hat Subjektivierung zur Folge. Die Anderen besinnen sich auf die Differenzen, die sie als anders markieren, und bestätigen diese oder widersprechen ihnen durch diskursive Selbstverortung. Othering-Diskurse erzeugen subalterne Subjektpositionen [32], indem sie Eindeutigkeit durch Dichotomisierung erzwingen. Ein ‚Flüchtling‘, der nicht als Opfer angesehen wird, gilt nicht als *echter* ‚Flüchtling‘ [49]. Das Bild eines *echten* ‚Flüchtlings‘ ist diskursiv geprägt und verweist auf ein weiblich konnotiertes, passives und hilfloses Opfer, das bescheiden, dankbar und anpassungsbereit ist. Eigenschaften wie Aktivität, Durchsetzungsvermögen, Forderungen und Tatkraft stehen im Widerspruch zum vorherrschenden Opferbild und ähneln eher dem des bedrohlichen Eindringlings [49].

*Othering führt zu einer imaginären Trennung zwischen einem Innen und Außen.*

Die imaginäre Trennung zwischen einem Innen und Außen kann sozialräumliche Auswirkungen haben und Projektionen ins Außen ermöglichen. Dies zeigt sich beispielsweise daran, dass Ereignisse im Zusammenhang mit ‚Migranten‘ häufig unter Berufung auf Kategorien, welche ihre Andersheit bestimmen (ethnische, kulturelle oder religiöse Unterschiede), thematisiert und als Probleme zwischen Innen und Außen artikuliert werden [28]. Dadurch wird ihre Andersheit immer wieder bestätigt und komplexe Strukturen – wie gesellschaftliche, ökonomische, politische sowie diskriminierende und rassistische – geraten aus dem Blickfeld. Gesellschaftlich verwurzelte Konflikte oder Phänomene können auf diese Weise wirkungsvoll zum Problem der Anderen gemacht werden, indem sie nach Außen und somit auf die Anderen projiziert werden (projektiver Umgang). Die gesellschaftlichen Zusammenhänge bleiben dabei verborgen. Vereinfacht ausgedrückt ist Othering deshalb so wirksam, weil es die Komplexität struktureller Problemlagen ausblendet.

*Othering kann sich sowohl in einer ablehnenden Absicht (z.* *B. durch Kriminalisierung, Dämonisierung) als auch in einer wohlmeinenden oder idealisierenden (z.* *B. Viktimisierung, Vulnerabilisierung, Exotisierung) äußern.*

Beide Formen des Othering entstammen einer Machtstruktur und festigen diese [50]. ‚Geflüchtete‘ als Opfer oder Kriminelle darzustellen, bleibt Teil einer dominanten Diskursstruktur. Auch in einer viktimisierenden Repräsentation werden ‚Geflüchtete‘ zu Objekten einer hegemonialen Interpretation, die in derselben machtvollen Dreiecksstruktur „Täter-Opfer-Retter“ [49] verharrt. In der Dreiecksbeziehung „Täter-Opfer-Retter“ nimmt das Nicht-Andere entweder die Rolle des *Retters* (der ‚Flüchtlinge‘, die aus *echter* Not fliehen und verfolgt werden) oder des *Opfers* (z. B. von Asylmissbrauch durch *kriminelle* ‚Flüchtlinge‘) ein, nie jedoch die des *Täters *[49]. Im sog. Flüchtlingsdiskurs kann sich das Nicht-Andere durch wohlmeinendes oder ablehnendes Othering als *Retter* oder *Opfer* imaginieren. Beide Interpretationen prägten die sog. Flüchtlingskrise 2015/2016.

*Othering ist ein kontingentes, kein notwendiges Phänomen.*

Othering erzeugt nicht nur Andere durch *eindeutige* Unterschiede, sondern beinhaltet auch die epistemische Macht, diese Unterschiede als Realität darzustellen. Jedoch verfügen die Anderen auch über ein hohes Potenzial zur Irritation. Sie schaffen Mehrdeutigkeiten, wie beispielsweise Mehrfachzugehörigkeiten. Aufgrund dieser Ambiguität und Unbestimmtheit können die Anderen die festgelegten Grenzen des Wir infrage stellen und somit die sozial konstruierte Realität des Wir relativieren. Daraus ergibt sich die Möglichkeit für einen konstruktiven Umgang mit Ambiguität: Durch die Irritationen, die der Andere hervorruft, entsteht das Potenzial für Dekonstruktionen.

Ausgehend davon zeichnen sich für Public Health künftig zwei Herausforderungen ab:

- erstens die Notwendigkeit, Othering erzeugende Denkweisen und Ordnungsstrukturen in der Gesundheitspraxis zu hinterfragen und neu zu gestalten,
- zweitens die Dringlichkeit, in der Gesundheitsforschung das durch Othering erzeugte Wissen über die Anderen zu dekonstruieren und seine Konsequenzen zu reduzieren.

## Footnotes

1 In dieser Arbeit verwenden wir einfache Anführungszeichen, um zu betonen, dass die verwendeten Begriffe als diskursive Konstrukte zu verstehen sind. Das heißt, nicht ‚Migranten‘ oder ‚Geflüchtete‘ als Subjekte stehen im Fokus der vorliegenden Arbeit, sondern ihre Wirkungsweise als diskursiv virulente Kategorien. Dementsprechend wird auf das Gendern dieser Begriffe zugunsten der Hervorhebung ihres kategorialen Charakters verzichtet.

2 In der Public-Health-Literatur wird Othering in inkludierende und exkludierende Formen unterteilt [6]. Wir haben diese Definition bereits in früheren Arbeiten [7, 8] kritisch analysiert und werden sie daher in dem aktuellen Artikel nicht weiter diskutieren.

3 Die methodische Vorgehensweise basiert auf einer theoretischen Konzeptionalisierung und impliziert eine analytische Synthese, die verschiedene Theorien und Konzepte aus unterschiedlichen Quellen/Disziplinen kombiniert, die im Vorfeld als relevant für die Beschreibung der Wirkungsweise des Othering identifiziert wurden.

4 Hall bezeichnet diese diskursiv hergestellte dualistische Trennung als eine Trennung zwischen dem Zentrum und der Peripherie [31] und meint damit eine Trennung zwischen dem ‚Westen‘ und dem ‚Rest‘ [40]. Nach Auffassung von Said [15] verläuft diese diskursive Trennung zwischen ‚Orient‘ und ‚Okzident‘.
